# A Novel Approach to Double the Sensitivity of Polarization Maintaining Interferometric Fiber Optic Gyroscope

**DOI:** 10.3390/s20133762

**Published:** 2020-07-05

**Authors:** Dengwei Zhang, Cui Liang, Nan Li

**Affiliations:** State Key Laboratory of Modern Optical Instrumentation, College of Optical Science and Engineering, Zhejiang University, Hangzhou 310027, China; seaskyzdw@zju.edu.cn (D.Z.); cui_liang@zju.edu.cn (C.L.)

**Keywords:** polarization maintaining interferometric fiber optic gyroscope, double sensitivity, fiber polarization combiner/splitter

## Abstract

In this paper, a novel optical approach to double the sensitivity to angular rate of interferometric fiber optic gyroscope (IFOG) is proposed. Two fiber polarization combiner/splitters (FPCSs), as the key components, are added in the traditional IFOG light path. The FPCSs are able to either combine two orthogonal polarizations transmitting at two different polarization-maintaining fibers (PMFs) into the two orthogonal axes of one PMF, respectively, or split two polarizations transmitting at the two orthogonal axes of one PMF into two polarizations to transmit at two different PMFs, respectively. Through the specific placement and coupling of these two FPCSs, the incident light can transmit twice along the polarization-maintaining fiber coil (PMFC). The novel approach is verified experimentally and the experimental results show consistency with the theoretical analysis. The proposed approach is able to double the sensitivity of IFOGs and can increase the signal-to-noise ratio (SNR) without increasing the length of PMFC, which is very susceptible to environmental influences and is of great significance in the technical improvement of IFOGs, as well as the miniaturization of IFOGs.

## 1. Introduction

The inertial navigation system (INS), as an autonomous navigation device that does not depend on external information, is able to provide continuous and real-time information, such as the position, attitude, and speed of the carrier. It has the advantages of good concealment and high precision, and is not affected by meteorological conditions and human disturbances as well [1,2,3,4,5]. Therefore, INS occupy an extremely important position in military technology, including aircraft navigation and weapon guidance. With the maturity of related technology, INS, especially high-precision INS, has been gradually applicated to aerospace, aviation, oil exploration, geodesy, automotive industry, medical electronic equipment, and other fields, showing an increasingly great role in these fields [6,7,8]. As the core devices in INSs, the performance of gyroscopes has great influences on navigation results. High-precision INSs require gyroscopes with high sensitivity. Interferometric fiber-optic gyroscopes (IFOGs) have the advantages of high theoretical sensitivity, all solid state, high reliability, low cost, and so on. Therefore, IFOGs, as the first choice in high-precision INS, have been widely used in both civil and military fields. In some applications, such as long-distance, long-duration navigation, high-precision IFOGs with low bias stability and low random walk are required [9,10,11,12,13]. However, improving the sensitivity is the basis for achieving high-precision IFOGs.

Numerous achievements have been made out to improve the sensitivity of IFOGs, including software compensation and hardware optimization. Software compensation [14,15,16,17] mainly aims at eliminating the effects of external environmental factors, such as temperature, magnetic field, and vibration. As a result, the environmental adaptability of IFOGs will be improved. As for hardware optimization, light sources with higher stabilization and larger output power [18,19,20], optical devices with better polarization-maintaining ability [21], electrical devices with higher stability [22], and signal-detection circuits with higher precision [23,24] are normally adopted. However, with the technology of IFOGs maturing rapidly, the common approaches for improving the sensitivity of IFOGs have faced many difficulties, which means only small promotions can be achieved with quite a high cost. Besides, by increasing the length and the surrounding area of fiber coil, the nonreciprocal phase shift caused by the angular velocity of the carrier relative to the inertial space can be accumulated and the sensitivity of IFOG can be improved. However, there will be two fatal disadvantages. One is that the volume of IFOGs will increase significantly, while another is that the environmental adaptability of IFOGs will be greatly reduced. Theoretical studies have been carried out to reveal about mechanism how temperature [25,26], magnetic field [27,28,29], vibration [30], and the coupling effect of physical fields [31] affect the IFOGs’ performance. In a nutshell, increasing fiber length makes the asymmetric internal stress distribution in the fiber coil much more complicated, making it more susceptible to environmental factors, causing a quite large nonreciprocal phase error. Therefore, it is not feasible to improve the sensitivity of IFOGs by simply increasing the length and surrounding area of fiber coils. K. Zhou et al. proposed a fiber gyroscope with a double sensitivity employing a polarization splitter made with an evanescent coupling technology [32] and W. Wu et al. further improved the method by adding a faraday rotator in open-loop fiber-optic gyroscope [33]; however, there exist obvious coupling errors and losses in the evanescent coupling theory based polarization splitter.

In this paper, we propose a novel approach, which is able to effectively double the sensitivity by doubling the light paths without increasing the length of polarization-maintaining fiber coil (PMFC), which is the sensing coil in IFOGs. The corresponding demonstration was setup and the effectiveness of the novel approach was verified. The experimental results show consistency with the design analysis.

## 2. Working Principle

**Figure 1:**
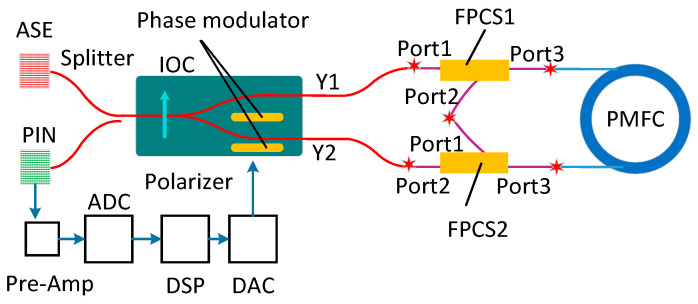
Schematic diagram of PM-IFOG with FPCS1 and FPCS2 inserting between PMFC and Y-waveguide, respectively. IOC, integrated optical chip; ASE, amplified spontaneous emission light source; ADC, analog-to-digital converter; DSP, digital signal processor; DAC, digital-to-analog converter; PIN, p-type, intrinsic, n-type photodiode.

The diagram of the proposed method is shown in Figure 1, which includes light source (ASE), integrated optical chip (IOC), PMFC, two fiber polarization combiner/splitters (FPCSs), p-type, intrinsic, n-type photodiode (PIN), fiber splitter, and the related data sample system, as well as the data processing system. The IOC consists of a polarizer, Y-branch coupler, and phase modulator. FPCS has three ports: Port1, Port2, and Port3. The two FPCSs are inserted between two Y-waveguides of IOC and two ports of PMFC; meanwhile, Port2 of FPCS1 welding with Port1 of FPCS2. Light emitted from ASE is split into two beams of clockwise (CW) light and counter-clockwise (CCW) light by Y-waveguide. Then the two light beams are doubled in the non-reciprocal light path. The two doubled lights interfere at PIN. Based on the pre-Amp, analog-to-digital converter (ADC), digital signal processor (DSP), digital-to-analog converter (DAC), and closed-loop control, the doubled non-reciprocal phase, which is caused by regular rate, can be achieved.

**Figure 2:**
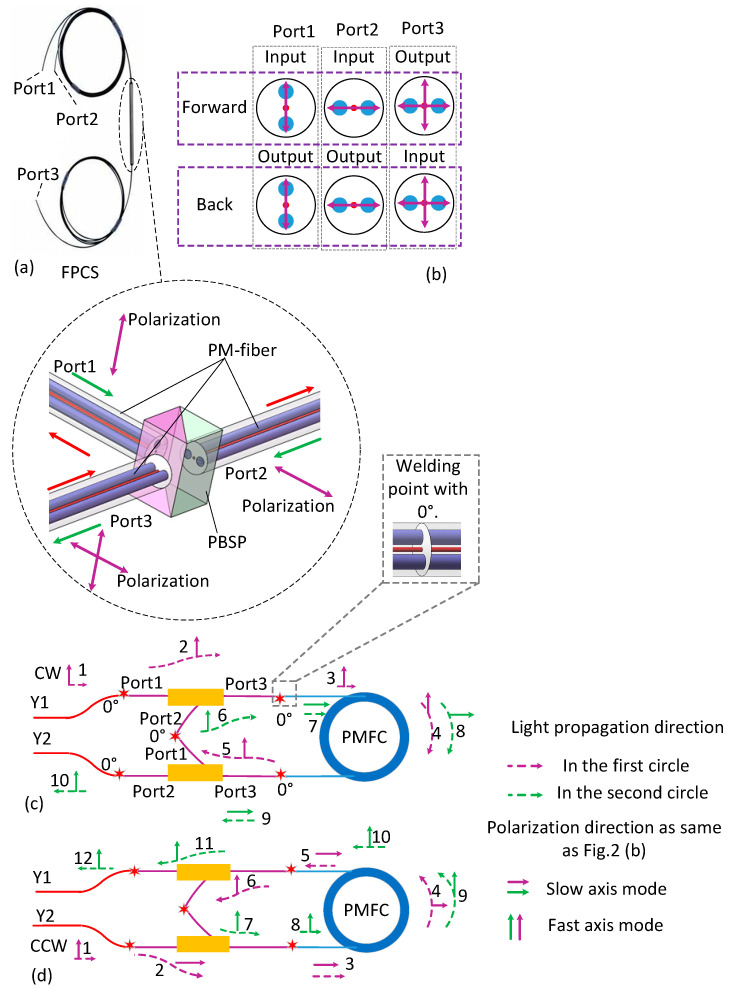
Working principle of FPCS and the proposed approach. (**a**) Schematic diagram of FPCS. FPCS-fiber polarization combiner/splitters; PBSP-polarization beam splitting prism. (**b**) The combining method of two polarization lights inputting from Port1 and Port2 to Port3, and the splitter method of one light including two polarizations transmitting vertically from Port3, then splitting two lights transmitting through Port1 and Port2, respectively. (**c**) The doubling approach of CW light that transmits from Y1 to Y2. (**d**) The doubling approach of CCW light that transmits from Y2 to Y1.

As shown in Figure 2a, the FPCS consists of one polarization beam splitting prism (PBSP) and three sections of polarization maintaining fiber (PMF), where the prism has two PMF branches on one side and one PMF branch on the other side. In the branch with two PMFs, the slow axis of the fiber is aligned with the maximum transmission direction of a polarization state.

The two polarizations transmit at two orthogonal axes, or split two polarizations, which transmit at two orthogonal axes in a single PMF, into its orthogonal linear polarizations through two fiber ports. Light only travels along a single axis, such as the slow-axis, of the PMF attached to Port1 and Port2, while it can travel along both the slow-axis and the fast-axis of the PMF attached to Port3, as shown in Figure 2a,b. The forward light beams aligned with the slow-axis of the two PMF branches are coupled into FPCS from Port1 and Port2. After passing through the prism, the light beam will be coupled into the two different axes of the PMF branch accordingly and get out from Port3. The polarization direction of the forward light beams, which are coupled into Port1 and Port2, should be aligned with the slow axis; otherwise, the light beams will be refracted into different directions and will not exit through Port3 finally. The back light beam is coupled from Port3. If the back light beam is aligned with the slow axis, it will be shifted by the prism and then exit from Port1 along the slow axis of the PMF attached to it. On the contrary, if the input light beam of Port3 is aligned with the fast axis, it will exit through Port2 along the slow axis of the PMF attached to it. The polarization extinction ratio of FPCS can reach 30 dB and the insertion loss will be less than 0.6 dB.

The light signal from the light source is converted into two linearly polarized light beams transmitted clockwise (CW) and counter-clockwise (CCW) by the Y-branch coupler, respectively. The CW light beam travels along the slow-axis of PMF attached to Port1 of FPCS1 and exits from its Port3 along the slow-axis of PMF attached to it. After one turn around the PMFC, the CW light beam enters the FPCS2 through its Port3 and get out from its Port1. The coupling angle of the PMFs attached to Port1 of FPCS2 and Port2 of FPCS1, is 0°. Therefore, the light beam enters FPCS1 again through Port2 and then exits from its Port3 along the fast-axis of PMF. After another turns around the PMFC, the CW light beam enters FPCS2 for the second time and exits from its Port2 along the slow-axis of the PMF. As for the CCW light beam, it travels along the slow-axis of PMF attached to Port2 of FPCS2 and exits from its Port3 along the fast-axis of PMF attached to it. After one turn around the PMFC, the CCW light beam enters FPCS2 through its Port3 and exits from its Port2. The coupling angle of the PMFs attached to Port1 of FPCS2 and Port2 of FPCS1 is 0°. Therefore, the light beam enters FPCS2 again through Port1 and then outputs through its Port3 along the slow-axis of PMF. After another turn around the PMFC, the CCW light beam enters FPCS1 for the second time and exits from its Port1 along the slow-axis of the PMF. Thus, because of the specific placement and coupling of these two FPCSs, the incident light can transmit twice along the PMFC, doubling the sensitivity of IFOG indeed.

## 3. Experimental Verification

**Figure 3:**
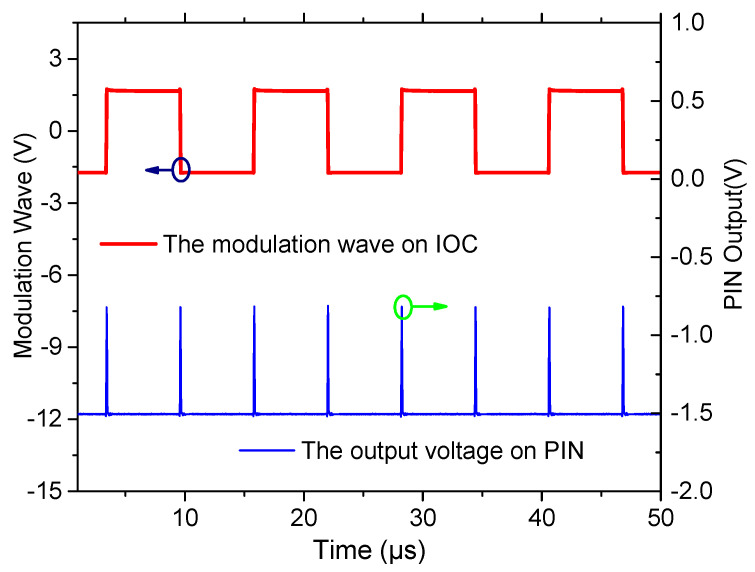
Measured time delay of the coil using square-wave modulation. PIN-p-type, intrinsic, n-type photodiode.

**Figure 4:**
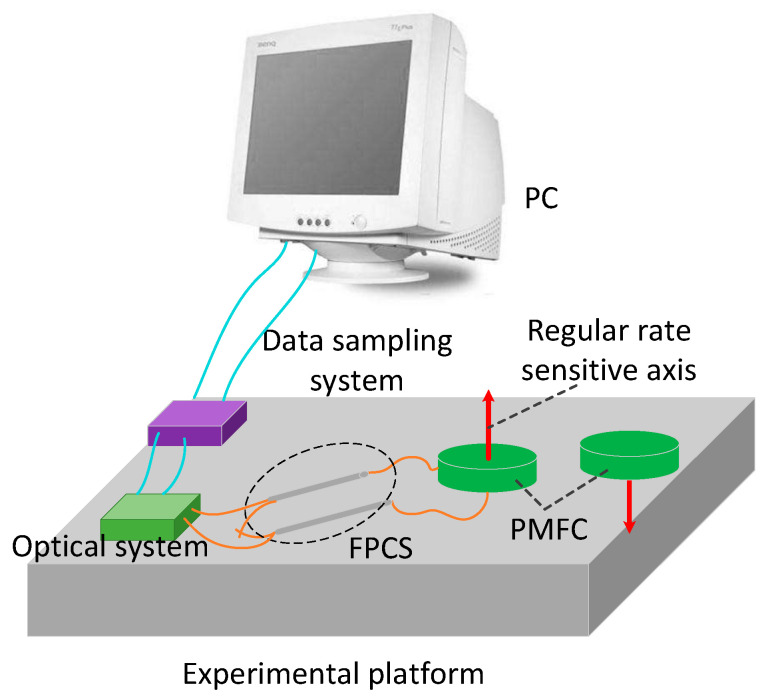
Experimental setup of the PM-IFOG before and after improvement for testing the angular rate of earth’s rotation. FPCS, fiber polarization combiner-/splitter.

**Figure 5:**
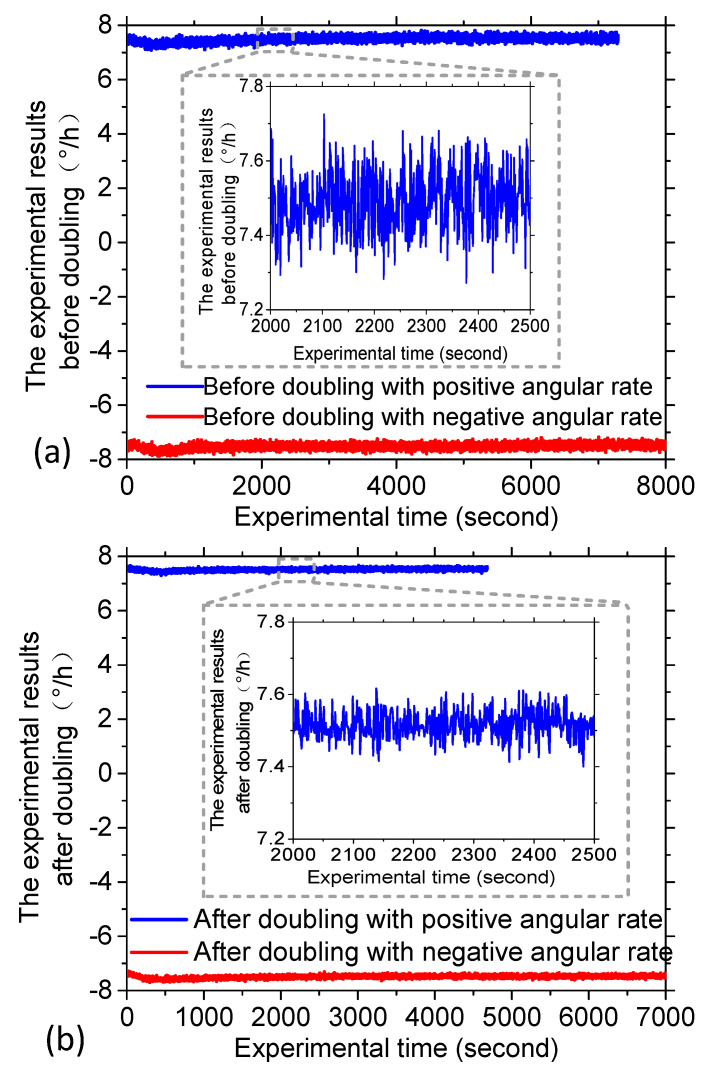
Experimental results. (**a**) The experimental results before doubling the sensitive optical paths with positive and negative input angular rate, respectively. (**b**) The experimental results after doubling the sensitive optical paths with positive and negative input angular rate, respectively.

**Figure 6:**
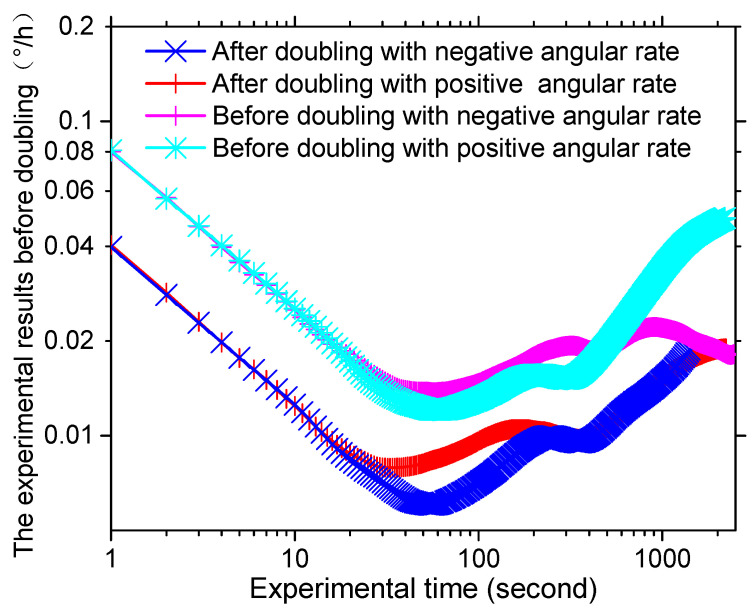
The Allan variances of PM-IFOG before and after doubling with positive and negative input angular rate, respectively.

In order to verify the effectiveness of the proposed method, the intrinsic frequency of the novel polarization maintaining interference fiber optical gyroscope (PM-IFOG) was measured. A square-wave voltage was applied to the phase modulator of the IOC. Tuning the frequency of the square wave, the delay time of the sensing coil for light wave after doubling is ~6.21 μs, as shown in Figure 3, which proved that the light waves traveled twice in the PM coil with the fiber length of 600 m and the coil diameter of 70 mm. To further prove its effectiveness, an experimental setup was established, as shown in Figure 4. Two FPCSs (Thorlabs) were added in a conventional PM-IFOG. Both the optical system and the data sampling system were fixed on the experimental platform. The PM-IFOG before and after improvement were used to detect the earth’s rotation angular rate at the same place, respectively. Before doubling the optical path, the PM-IFOG, the length of whose PMFC is 600 m, were put on the experimental platform with the sensing axis straight up and straight down, respectively. The corresponding input regular rates were positive and negative and equal to the component of the earth’s rotate rate. As we know, the averaged output angular rates of earth’s rotation are 15°/h and −15°/h. It is well known that the theoretical value of positive and negative (Hangzhou, China) where the experiments were carried out lies at a latitude of ~$30^{{}^{\circ}}\;N$, so the actual value of positive and negative angular rate of earth’s rotation in the experiments were supposed to be 7.5°/h and −7.5°/h. The output data, D_1_, with the input regular rate of 7.5°/h and D_2_ with the input regular rate of −7.5°/h of PM-IFOG at the two conditions, were collected in real-time and then saved to the PC. The scale factor can be calculated as K = (D_1_ − D_2_)/15°/h. The experimental result data divided by K on the two conditions are shown in Figure 5a. After doubling the optical path, the same experiments were done and the processed experimental results are shown as Figure 5b. The positive and negative angular rate value of the normal PM-IFOG before doubling were 7.4895°/h and −7.5105°/h, while the averaged output of IFOG after doubling became 7.51258°/h and −7.48744°/h accordingly. From the insets of Figure 5a,b, we can see the signal-to-noise ratio (SNR) improved greatly after doubling the light path. The Allan errors of the experimental results were analyzed and shown in Figure 6.

Based on the noise analysis theory for gyroscopes, angle random walk (ARW) appears where the slope of Allan error curve is −0.5 and bias stability is thought to be the minimum value of the curve where the slope is 0 divided by 0.664 [34]. The values of ARW and bias stability of the IFOGs before and after doubling were calculated according to the method mentioned above and are shown in Table 1. It was obvious that the ARW, after doubling the light path, was relatively smaller than conventional PM-IFOG, which means a higher SNR. Meanwhile, compared with the conventional PM-IFOG, the positive and negative sensitivity of PM-IFOG after doubling the light path was promoted to be 2.002 and 1.740 times, according to the promotion of bias stability. In addition, the bias stability with the positive earth rotate angular rate after doubling was 0.0091°/h. Compared to Ref. [32], we have achieved high precision gyro prototype with miniaturization.

**Figure 7:**
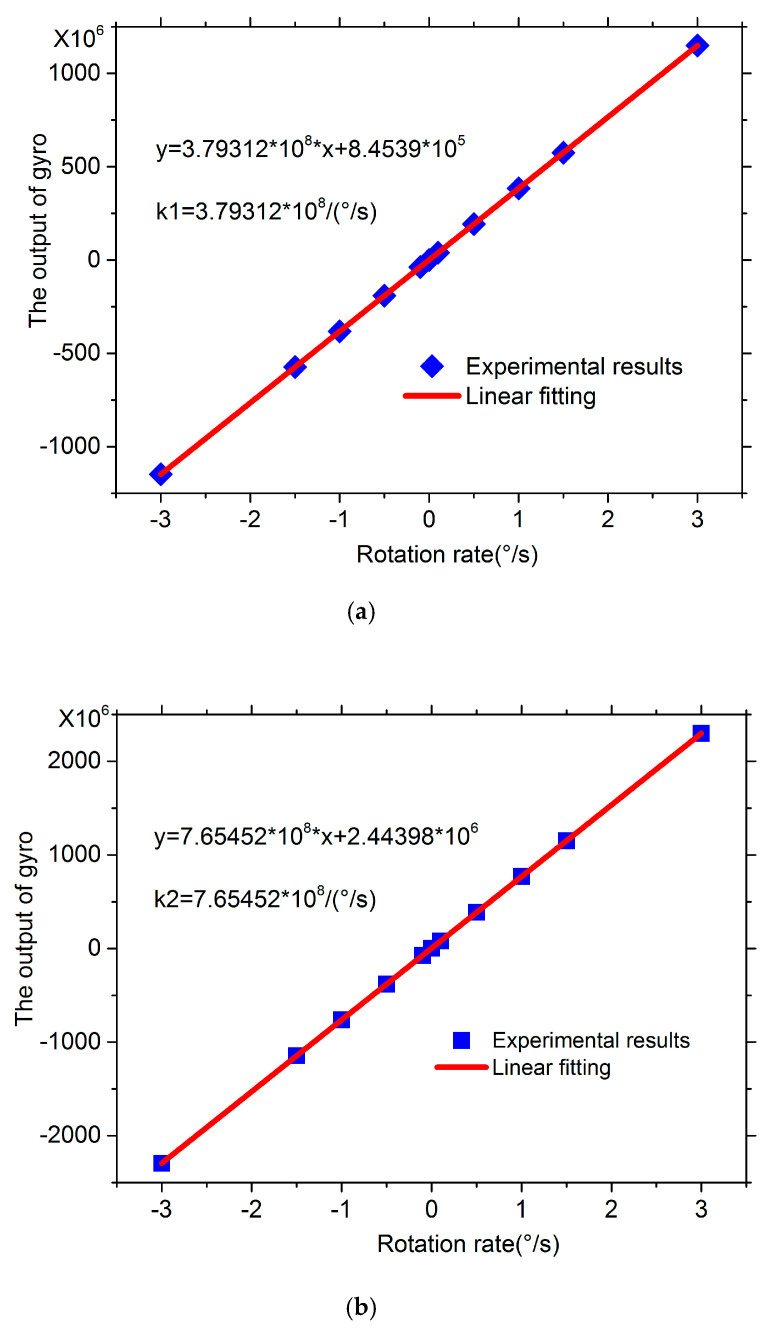
Scaling factor experimental results with the rotation rate from −3°/s to 3°/s. (**a**) Before doubling. (**b**) After doubling.

The demonstrations before and after doubling were placed on a motor-driven turntable with the rotation rates of −3°/s and 3°/s, respectively. The scaling factor experimental results are shown in Figure 7a,b, respectively. Before doubling, the scaling factor k1 with the rotate rate from −3°/s to 3°/s was 3.79312 × 10^8^/(°/s), but after doubling, the scaling factor k2 was 7.65452 × 10^8^/(°/s). The doubling factor was k2/k1 = 2.018. Compared to the expected value 2, the experimental doubling factor was 1% larger. This may lead by the pigtails of the two FPCSs, each of whose length is ~1.5 m.

## 4. Conclusions

IFOGs are widely used due to their excellent performance and are the core component of many devices including high-precision INS. Improvement of the sensitivity of IFOGs is extremely required in some specific applications. A novel approach for doubling the sensitivity of PM-IFOG is proposed. Two FPCSs were added in a conventional PM-IFOG system in a specific way of placement and coupling. In the process of detecting the earth’s rotation angular velocity, experimental results showed that the sensitivity of PM-IFOG with the novel approach indeed was basically twice that of the normal PM-IFOG, which was consistent with the theoretical analysis, and thus verified the validity of the proposed method. Meanwhile, the SNR of the PM-IFOG with novel approach also obtains a promotion. In conclusion, the PM-IFOG with the proposed doubling method has the obvious advantages of higher sensitivity and constant environmental adaptability, which is quite meaningful in engineering applications, especially in the field requiring long fiber coils.

## Figures and Tables

**Table 1:** Calculated sensitivity of the PM-IFOG when sensing earth’s rotation angular rate.

	Positive before Improvement	Negative before Improvement	Positive after Improvement	Negative after Improvement
Theoretical data (°/h)	7.5	−7.5	7.5	−7.5
Average data acquired (°/h)	7.4895	−7.5105	7.5126	−7.4874
Angle random walk (${}^{{}^{\circ}}/\sqrt{h}\;\;$)	0.0013	0.0013	0.0007	0.0007
Bias stability (°/h)	0.0182	0.0208	0.0091	0.0120

## References

[1] Groves P.D. (2013). Principles of GNSS, Inertial, and Multi-sensor Integrated Navigation Systems. Ind. Robot.

[2] King A.D. (1998). Inertial Navigation—Forty Years of Evolution. GEC Rev..

[3] Yan Z., Chen X., Tang X. (2020). A Novel Linear Model Based on Code Approximation for GNSS/INS Ultra-Tight Integration System. Sensors.

[4] Chang L., Hu B., Li A., Qin F. (2013). Strapdown inertial navigation system alignment based on marginalised unscented Kalman filter. IET Sci. Meas. Technol..

[5] Castaneda N., Lamy-Perbal S. An improved shoe-mounted inertial navigation system. Proceedings of the 2010 International Conference on Indoor Positioning and Indoor Navigation.

[6] Braden K., Browning C., Gelderloos H., Smith F., Marttila C., Vallot L. Integrated inertial navigation system/Global Positioning System (INS/GPS) for manned return vehicle autoland application. Proceedings of the IEEE Symposium on Position Location and Navigation. A Decade of Excellence in the Navigation Sciences.

[7] Tucek J., Kardos M., Tomastik J. (2016). First experience with pedestrian Inertial Navigation System application under forest conditions. Zpravy Lesnickeho Vyzkumu.

[8] Stelkens-Kobsch T.H. (2006). Further Development of a High Precision Two-Frame Inertial Navigation System for Application in Airborne Gravimetry. Observation of the Earth System from Space.

[9] Wang H.G., Williams T.C. (2008). Strategic inertial navigation systems—High-accuracy inertially stabilized platforms for hostile environments. Control Syst. IEEE.

[10] Ahrens S., Levine D., Andrews G., How J.P. Vision-based guidance and control of a hovering vehicle in unknown, GPS-denied environments. Proceedings of the 2009 IEEE International Conference on Robotics and Automation.

[11] Shkel A.M. Precision navigation and timing enabled by microtechnology: Are we there yet?. Proceedings of the IEEE Sensors 2010 Conference.

[12] Zhou Y., Lai J., Guo X., Yang J. A research on all source navigation and positioning and its critical technology. Proceedings of the 6th China Satellite Navigation Conference.

[13] Wang L., Zhang C. (2006). Modeling of FOG and application in inertial navigation system. J. Chin. Inert. Technol..

[14] Zhang D., Zhao Y., Zhou W., Fu W., Liu C., Shu X., Che S. (2014). A software-compensation method to orthogonal magnetic field drift in a depolarized fiber-optic gyroscope. Optik.

[15] Dranitsyna E.V., Egorov D.A., Untilov A.A., Deineka G.B., Sharkov I.A., Deineka I.G. (2013). Reducing the effect of temperature variations on FOG output signal. Gyrosc. Navig. B.

[16] Feng L.S., Nan S.Z., Jin J. (2006). Research on modeling and compensation technology for temperature errors of FOG. J. Astronaut..

[17] Chen X., Shen C. (2013). Study on temperature error processing technique for fiber optic gyroscope. Optik.

[18] Moslehi B., Yahalom R., Oblea L., Faridian F., Black R.J., Ha J.C., Berarducci M. Low-cost and compact fiber-optic gyroscope with long-term stability. Proceedings of the 2011 Aerospace Conference.

[19] Su H.C., Wang L.A. (2003). A highly efficient polarized superfluorescent fiber source for fiber-optic gyroscope applications. IEEE Photonics Technol. Lett..

[20] Li X.Y., Zhang R.P., Wu L., Zhang Y.G. (2010). Suppression for light source intensity noise in high-precision FOG. J. Chin. Inert. Technol..

[21] Kintner E.C. (1981). Polarization control in optical-fiber gyroscopes. Opt. Lett..

[22] Chen X., Yang J.H., Zhou Y.L., Shu X. (2018). The application of low-noise DC-DC power source in fiber-optic gyroscope system. Opto-Electron. Eng..

[23] Toyama K., Fesler K.A., Kim B.Y., Shaw H.J. (1991). Digital integrating fiber-optic gyroscope with electronic phase tracking. Opt. Lett..

[24] Wang Q., Yang C., Wang X., Wang Z. (2013). All-digital signal-processing open-loop fiber-optic gyroscope with enlarged dynamic range. Opt. Lett..

[25] Shupe D.M. (1980). Thermally induced nonreciprocity in the fiber-optic interferometer. Appl. Opt..

[26] Mohr F. (2002). Thermooptically induced bias drift in fiber optical Sagnac interferometers. J. Lightwave Technol..

[27] Hotate K., Tabe K. (1986). Drift of an optical fiber gyroscope caused by the Faraday effect: Influence of the earth’s magnetic field. Appl. Opt..

[28] Liu P., Li X., Guang X., Li G., Guan L. (2017). Bias Error Caused by the Faraday Effect in Fiber Optical Gyroscope with Double Sensitivity. IEEE Photonics Technol. Lett..

[29] Hotate K., Saida T. (1999). General formula describing drift of interferometer fiber-optic gyro due to Faraday effect: Reduction of the drift in twin-depo-I-FOG. J. Lightwave Technol..

[30] Song N. (2004). Analysis of vibration error in digital closed-loop fiber optic gyroscope. J. Beijing Univ. Aeronaut. Astronaut..

[31] Liang C., Zhang D., Zhou Y., Shu X., Che S., Liu C. (2019). Coupling effect of a single-mode fiber coil under time-varying temperature and magnetic field. J. Lightwave Technol..

[32] Zhou K., Pan S., Liu S., Hu K. (2013). Fiber gyroscope with a double sensitivity employing a polarization splitter. Opt. Lett..

[33] Wu W., Zhou K., Lu C., Xian T. (2018). Open-loop fiber-optic gyroscope with a double sensitivity employing a polarization splitter and Faraday rotator mirror. Opt. Lett..

[34] Board I. (1998). IEEE Standard Specification Format Guide and Test Procedure for Single-Axis Interferometric Fiber Optic Gyros.

